# The interplay between internal communication, employee engagement, job satisfaction, and employee loyalty in higher education institutions in Vietnam

**DOI:** 10.1057/s41599-023-01806-8

**Published:** 2023-06-15

**Authors:** Cao Minh Anh Nguyen, Minh-Tri Ha

**Affiliations:** 1 Becamex Business School, Eastern International University, Thu Dau Mot City, Vietnam; 2 School of Business, International University, VNU-HCM, Ho Chi Minh City, Vietnam; 3 Vietnam National University—Ho Chi Minh City, Ho Chi Minh City, Vietnam

**Keywords:** Business and management, Education

## Abstract

The present study examines the roles of internal communication (IC), job engagement (JE), organisation engagement (OE) and job satisfaction (JS) in producing employee loyalty (EL) based on the social exchange theory. This study employed an online questionnaire-based survey design to collect data from 255 respondents from higher education institutions (HEIs) in Binh Duong province using convenience and snowball sampling techniques. Data analyses and hypothesis testing were carried out using the partial least squares structural equation modelling (PLS-SEM). The findings show that all relationships are significantly validated, except for the JE-JS relationship. Our work is the first to investigate employee loyalty in the HEI context of an emerging economy such as Vietnam by incorporating internal communication, employee engagement (including job and organisation engagement) and job satisfaction to develop and validate a research model for the study. This study is expected to contribute to the theory and advance our understanding of different mechanisms that job engagement, organisation engagement and job satisfaction can play in the relationship between internal communication and employee loyalty.

## Introduction

Employee loyalty (EL) plays a crucial role in deciding an organisation’s success. Masakure (2016) defines it as the positive attitudes and behaviour of employees towards their employers or workplace. Moreover, Lee et al. (2013) believe that highly loyal employees tend to remain in their roles and be satisfied with them. Prior studies indicate that several elements can lead to employee loyalty in the workplace such as employee engagement (EE), job satisfaction (JS), compensation, benefits, or even effective internal communication (IC) strategies (Abror et al. 2020). In a similar fashion, this also happens in higher education institutions, where education is a core value for contributing to the development of society and the nation. In recent years, the competition in this field is tough. Each higher education institutions needs to improve its quality of study programs, technology applications, or infrastructure to attract students compared to other competitors (Asrar-ul-Haq et al. 2017). Instead of achieving these challenges, human resources (HR) is a crucial element for developing the quality of any higher education institution in several factors, especially faculty and non-academic staff (Abror et al. 2020). This means that it is a valuable asset in any organisation. To improve human resources in the workplace, job satisfaction is a variable that the employer should consider. It refers to negative or positive feelings of individuals about their job (Asrar-ul-Haq et al. 2017). Previous research highlights that having more satisfied employees in the company leads to a higher competitive advantage. Moreover, it also affects reputation and performance as a whole (Abror et al. 2020). Generally, several empirical pieces of evidence identify the significant relationship between job satisfaction and employee loyalty in different sectors such as public health (Vuong et al. 2021), banking (Hassan et al. 2013), energy (Matzler and Renzl 2006), and lodging (Kim and Vinh 2020).

In addition to job satisfaction, internal communication, and employee engagement are also mentioned in the present study. Internal communication is a strategy for connecting employers and employees, because both parties can easily exchange important information (Mishra et al. 2014). It is an essential tool to motivate employees in different aspects. Mandal and Gunasekaran (2003) believe that a successful internal communication system increases the positivity of employees’ attitudes and behaviour to achieve work outcomes. With effective internal communication, employees enhance performance and gain trust in the organisation (Nadeak and Naibaho 2020), which means that this construct plays an indispensable role in human resource practices and should be implemented in several departments (Ahmed et al. 2003). Therefore, its function is as important as external communication with customers (Dwairi et al. 2007). Employee engagement is described as “the degree to which an individual is attentive and absorbed in the performance of their roles” (Saks 2006, p. 602). Previous research points out that, if managers implement effective and suitable internal communication tools, employees tend to be more engaged with their job and workplace (Jacobs et al. 2016). Moreover, internal communication also impacts productivity, trust, and even commitment (Pounsford 2007). On the other hand, internal communication also impacts employee loyalty in the organisation. Loyalty in employees is built up based on different elements such as internal communication. The effective system helps employees to know about new announcements, regulations, procedures, and important events. It makes them more engaged and loyal (Narteh and Odoom 2015). In the banking sector, timely information sharing improves employee performance, productivity, and management decisions leading to loyalty in the working environment (Narteh and Odoom 2015). However, in the educational sector, the loyalty level of academic and non-academic staff is positively affected by internal communication through motivation. This means that motivation is a mediating factor in the relationship between internal communication and employee loyalty (Nadeak and Naibaho 2020).

In 2019, the Vietnamese Government enacted Acts relating to education to attract international and national talents to improve human resource quality in higher education institutions. Up to the time of writing, there have been several positive changes. For instance, eight Vietnamese universities were ranked in the top 500 Best Asian Universities in 2020. High educational quality also contributes to the Human Quality Index. However, educational experts have identified some challenges leading to disloyal employees, and that ineffective internal communication affects unclear management and decreases the level of employee engagement and job satisfaction (Bindl and Parker 2010; Mishra et al. 2014). This indicates the importance of internal communication in improving different aspects of employees’ well-being. Previous academics have demonstrated the relationship between these variables in several industries such as hospitality (Lee et al. 2014), banking (Hassan et al. 2013), and manufacturing (Verčič and Vokić 2017). However, none has ever addressed internal communication and employee loyalty in the context of higher education institutions post-COVID-19. The present study will examine this research model in Vietnamese higher education institutions (HEIs), particularly universities in Binh Duong. Binh Duong province was selected for our study due to the following three reasons:

1. Regional focus: Binh Duong is one of the fastest-growing provinces in Vietnam, with rapid industrialisation and urbanisation (Mai et al. 2023). This makes it an interesting case study in terms of regional focus for understanding how HEIs in the region are evolving to meet the growing demands of industry and the labour market.
2. Manageable scope: studying higher education in the entire country can be an enormous task, given the sheer number of institutions and diverse regional characteristics. Focusing on a specific region or province, such as Binh Duong, allows for a more manageable scope and depth of analysis.
3. Policy implications: Binh Duong’s development strategies may offer valuable insights for other regions in Vietnam and beyond. Studying HEI in this context could provide policy recommendations for enhancing the quality and relevance of higher education to support economic growth and social development.

Moreover, because of post-COVID-19 and tough competition within schools, they also need to enhance their reputation as well as attract more students. Therefore, retaining highly skilled employees is one of the top priorities in any organisations. Furthermore, this study’s results can be a reliable source for university administrators to decide which strategies need to be implemented to retain employees and raise their reputations. The mediating role of employee engagement, including job engagement (OE) and organisation engagement (OE) in the internal communication-job satisfaction linkage will also be considered. On the other hand, this research is one of the first studies investigating Vietnamese higher education institutions.

This study offers two novelties: first, this study is the first study post-COVID-19 that examines employee loyalty by applying the social exchange theory in the higher education institutions context of an emerging economy such as Vietnam by incorporating internal communication, employee engagement (including job and organisation engagement) and job satisfaction to develop a research model for the study. This study is expected to contribute to the theory and advance our understanding of different mechanisms that job engagement, organisation engagement, and job satisfaction can play in the internal communication and employee loyalty relationship. Second, this very rare research examines the mediating role of employee engagement conceptualised in both job engagement and organisation engagement simultaneously in the relationship between internal communication and employee loyalty in the higher education institutions setting. Prior research validated employee engagement in different sectors such as banking (Karatepe and Aga 2012), information technology (IT) (Riyanto et al. 2021), hospitality (Lee et al. 2014; George et al. 2020), enterprise sector (Yao et al. 2020; Leung 2008; Pace and Kisamore 2017), healthcare sector (Jankelová et al. 2021) and public service organisations (Coyle-Shapiro et al. 2006), except the higher education institutions setting. The findings enrich employee loyalty literature in the higher education institutions context of an emerging economy like Vietnam.

To address these research gaps, this study attempts to answer the following research questions:

1. What are the roles of internal communication, job engagement, organisation engagement, and job satisfaction in producing employee loyalty?
2. Is job engagement mediating the internal communication-job satisfaction and internal communication-employee loyalty relationships?
3. Is organisation engagement mediating the internal communication-job satisfaction and internal communication-employee loyalty relationships?

## Literature review and development of hypotheses

### Social exchange theory (SET)

Blau (1964) describes SET as the exchange of reciprocity to explain individuals’ behaviours and attitudes in different settings, especially in working situations which are known as the rule of payment. Moreover, this theory explains the effect of EE’s level on JS or EL in the workplace (Saks 2006). This rule shows that, in the working context, people tend to compare the outcomes and benefits (rewards) which they can each receive after finishing. They decide whether they are satisfied or not based on that (Macdonald and MacIntyre 1997). As mentioned above, the engagement level is explained by the exchange rule. If the organisation provides necessary resources, suitable regulations, attractive rewards, and benefits, employees are more engaged in their job, and vice versa (Saks 2006). Another point worth noting is that SET also clarifies the implementation of IC in the workplace. When applying an effective IC system to announce important decisions or information, mutual understanding and relationships in the working environment would increase as well as employee engagement. This means that applying SET in this study is reasonable and it explains the relationships between constructs.

### Internal communication

Internal branding is a very popular term in management and it is applied in several workplaces to help managers link employees’ values and organisational goals (Foster et al. 2010). Individuals can easily understand the mission and vision of the company through internal branding (Choi 2006). Moreover, effective internal branding should connect employees’ work with organisational objectives and maintain the relationship between these parties (Punjaisri et al. 2009). In the paper of Lee et al. (2014), researchers note that IC is one of the elements of internal branding besides training and reward. Communication helps individuals to understand their role and deliver the company’s values to outsiders (Harris and de Chernatony 2001). Effective communication increases trust in employees and job performance even if they receive low compensation (Punjaisri et al. 2009). In recent years, communication has become one of the most interesting topics being explored by researchers, especially in the management field (Slijepčević et al. 2018). Generally, IC focuses on the exchange of information between the social actors (i.e. employers and employees) in the working environment (Cornelissen 2011). IC is defined by several academics. Dolphin (2005) states that IC is a system to connect people at work at different levels and functions. It plays a powerful role in public relations in huge companies (Kreps 1989). Moreover, the study by Meng and Berger (2019) suggests that, by utilising this tool, the manager can easily spread important information, improve relationships with employees and develop an organisational culture within the organisation. Meng and Berger (2019) also say that IC creates a friendly and professional environment for individuals in the working environment. Jacobs et al. (2016) suggest the announcement of new changes, policies, or regulations to employees within the organisation, including formal and informal activities. IC helps to improve employee morale, organisational culture, attitudes, and even individual behaviours (Ndlovu et al. 2021). In this study, Tkalac Vercic et al.’s (2012) definition will be adopted, in that IC is the process that spreads important announcements to enhance relationships, adopt new changes, and build up an understanding among employees. The vital role of IC in the working environment cannot be denied. Instead of increasing communication, it also leads to other organisational outcomes such as engagement, satisfaction, or even loyalty. The strengths and weaknesses of the organisation can be identified by applying IC. Several studies indicate that the effective system raises productivity, innovation, and the quality of work as well as decreases the turnover rate (Slijepčević et al. 2018). SET can be applied to explain the relationship between IC, EE, and JS in the present study. Based on the exchange of reciprocity, if the organisation provides enough resources and essential information to employees through IC, employees tend to engage in the job and workplace as well, which leads to raised satisfaction and loyalty (Punjaisri et al. 2009). Moreover, having a specific view of the operation and the value of the company also reduces misunderstandings in the working environment (King 2010). Tkalac Vercic et al. (2012) state that effective IC tools help employees to become more engaged with the organisation, which also leads to improved performance. Moreover, Lockwood (2007) says that IC is also the antecedent of EE which contributes to increasing employees’ engagement in both the job and the workplace.

Prior studies confirm the positive relationship between IC and EE, and executives in public relations observe that IC strategies improve the engagement level within the organisation (Mishra et al. 2014). The research in Croatia’s manufacturing industry has the same finding when investigating their employees (Verčič and Vokić 2017). According to Jacobs et al. (2016), when the association between IC and JS was investigated in China’s manufacturing industry, it indicated that transparent communication within the organisation can increase satisfaction and interaction between managers and employees. The result has been found by Nikolić et al. (2013) and Waters et al. (2013). Thus, this study will examine the relationship between these variables in Vietnamese HEIs.

Hypothesis 1a: Internal communication positively influences job satisfaction.

Hypothesis 1b: Internal communication positively impacts job engagement.

Hypothesis 1c: Internal communication positively influences organisational engagement.

### Employee engagement

Kahn (1990) describes EE as the commitment of employees in which they contribute themselves to their work and the organisation as an exchange of resources they have from the working environment. Moreover, Maslach et al. (2001, p. 74) propose this construct as “a positive fulfilling, work-related state of mind that is characterised by vigour, dedication and absorption”. The same study also refers to the importance of the connection between the company and its employees. Moreover, employees tend to have positive attitudes and behaviours at work and link their values with the organisation’s goals (Rothbard 2001). The study by Bedarkar and Pandita (2014) states that the definition of EE is defined as the relationship level between employers and employees in terms of physical, emotional, and cognitive development. EE improves the connection between an individual’s goals and the company’s objectives which avoids burnout, negative attitudes, and unethical behaviour in the workplace (Saleem et al. 2020). In terms of dimensions, researchers identify several types of EE in different studies. Rothbard (2001, p. 176) clarifies that attention and absorption are two major elements of EE. Attention is described as “cognitive availability and the amount of time one spends in thinking about a role” whereas absorption means “being engrossed in a role” and refers to “the intensity of one’s focus on a role”. Rothbard (2001) believes that researchers should focus on different roles of engagement within the organisation to ensure that they understand its impact on the workplace. In several studies, job engagement and organisation engagement are two other elements which are proposed by Kahn (1990) to examine engagement. According to Saks (2006), job engagement refers to the level of commitment and contribution to an employee position. Moreover, organisation engagement is defined as the level of loyalty towards the organisation (Saleem et al. 2020). In summary, several dimensions are discovered by different academics. In this study, job engagement and organisation engagement are adopted to test employee engagement. EE has various impacts on organisation outcomes based on several studies. High engagement leads to increasing positive behaviours, work performance, JS or commitment (Saks 2006; Chughtai and Buckley 2009). When enhancing engagement, employees also improve themselves in the workplace, for instance, in their motivation, creativity, morale and responsibility which affect their career path as well as organisational performance (Saleem et al. 2020).

Several studies have investigated the impact of EE on JS in different industries. Research studies in information technology and banking suggest that EE is the main element leading to high satisfaction in employees (Kamalanabhan et al. 2009). Moreover, after surveying non-academic staff in educational institutions in Thailand, engaged employees have a higher satisfaction level compared to others. This finding is supported by Chan et al. (2020), which was conducted based on education in Hong Kong. Different studies confirm the positive influence of EE and EL; after researching 389 faculties in Indonesia’s education, it is proven that loyal employees tend to be more engaged with their job and the organisation (Abror et al. 2020). The same result is confirmed by Book et al. (2019). To sum up, fewer studies examine the EE-JS and EE-EL linkage in education, and particularly in HEIs. In the present study, these relationships will be tested in the HEIs setting in Binh Duong, Vietnam. We propose the following hypotheses:

Hypothesis 2a: There is a positive relationship between job engagement and job satisfaction.

Hypothesis 2b: Job engagement positively influences employee loyalty.

Hypothesis 3a: Organisation engagement positively affects job satisfaction.

Hypothesis 3b: Organisation engagement positively impacts employee loyalty.

### Job satisfaction and employee loyalty

JS is one of the most popular topics and has been discovered in several industries. Ndlovu et al. (2021) state that individuals are willing to contribute themselves to the workplace if their employers offer fair compensation, benefits and suitable working conditions. Different definitions of JS are applied in previous studies; Tsai (2011) proposes that JS is the element to measure employee satisfaction including the characteristics or environments. On the other hand, JS also describes individuals’ emotions and affection towards their job (Judge et al. 1994). In this study, the definition of JS is adopted from Locke (1976), and refers to “a pleasurable or positive emotional state, resulting from the appraisal of one’s job or job experiences”. In terms of dimensions, Vroom (1962) states that superior, promotion, company, reward, working environment, job content and colleagues are the seven components of JS. Other dimensions are promotion, pay and work which are stated by Locke (1976). This study will apply the JS components of Driscoll (1978) including salary, promotion, security and decision-making.

Generally, JS consists of employees’ attitudes and behaviours in their roles and rewards, and internal and external factors such as society or the working environment. JS positively affects the satisfaction level (Leap and Crino 1993). In previous studies, JS impacts on performance, which means that satisfied employees tend to have a higher performance at work compared to others (Li et al. 2018). Moreover, company development and profit are influenced when employees achieve high satisfaction. Gu and Siu (2009, p. 564) say that “if a business wants to satisfy the needs of its customers, it must first satisfy the needs of its employees”. In an education environment, if teachers are satisfied, the education will contribute to students’ quality and school reputation as well (Li et al. 2018). Loyalty in employees is one of the main elements contributing to organisation development (Bloemer and Odekerken 2006). When individuals believe in the values and goals of the organisation, they will respond positively to attitudes and behaviours which increase employee retention (Cannon-Bowers et al. 1995). Moreover, Becker et al. (1995) define EL as “a willingness to slug on high levels of efforts for the sake of the organisation, and a definite belief in, and admissibility of, the values and goals of the organisation”. This construct refers to “the relative strength of an individual’s identification with, and involvement in, a particular organisation” (Wu and Norman 2006). In this study, the definition of EL is adopted from the explanation by (Becker et al. 1995). In terms of dimensions, different academics propose several elements of EL. Dick and Basu (1994) classify four types of loyalty which are no loyalty, spurious, latent and true loyalty. On the other hand, Byun et al. (2007) divide the variable into two main elements: behavioural and attitudinal loyalty. Attitudinal loyalty focuses on the attitudes of individuals towards the company (Broyles 2009). Jaiswal and Niraj (2011, p. 166) define it as “the cognitive basis of loyalty and isolates purchases driven by a strong attitude formed due to situational constraints”. This means that attitudinal loyalty is subjective and sometimes extremely difficult to measure, so several academics focus on behavioural loyalty rather than the other forms. In the present study, EL will focus on behavioural loyalty. Generally, loyalty is one of the most important factors in any organisation, and organisations with high loyalty lead to increased levels of success, survival and competitive advantages when compared to other competitors (Cooil et al. 2007). Moreover, managers also decrease turnover rates and additional costs such as training or recruiting (Vuong et al. 2021).

Previous studies examine the positive impact of JS on EL in several industries. After surveying doctors in hospitals in Vietnam’s public health system, researchers conclude that JS positively influences the level of EL (Vuong et al. 2021). This finding has the same result as a study conducted in Vietnam’s lodging industry. Individuals who are satisfied with their job tend to have an increased loyalty to the organisation (Kim and Vinh 2020). In other countries, this relationship has been assessed and conducted in public banks (Hassan et al. 2013) and the energy sector (Matzler and Renzl 2006). Hassan et al. (2013) believe that, if employees receive suitable compensation and benefits, their satisfaction and loyalty will both increase. Moreover, loyalty is the outcome of job satisfaction in management (Matzler and Renzl 2006). However, fewer studies have conducted this correlation in education, especially in Vietnam, so this study will examine it in Binh Duong, Vietnam. We propose the following hypothesis:

Hypothesis 4: Job satisfaction positively impacts employee loyalty.

### Employee engagement as a mediator

**Fig. 1:**
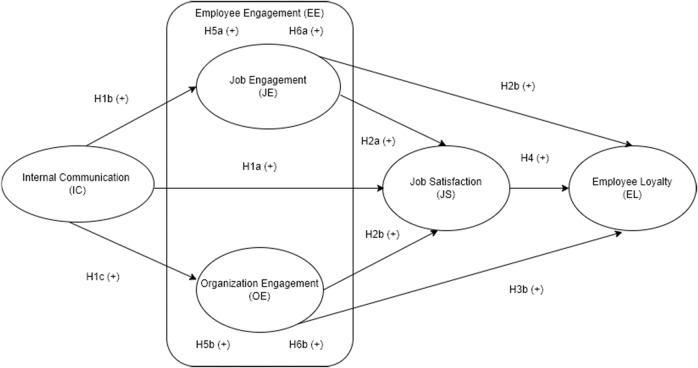
Hypothesised model.

The mediating role of EE in the relationship between organisational and personal outcomes has been proven in prior studies. Maslach et al. (2001) state that this variable mediates the association between working conditions and outcomes such as an internal branding, reward system, JS or EL. After surveying employees in Cyprus banks, researchers conclude that work engagement mediates the job resources and job satisfaction linkage (Karatepe and Aga 2012). Moreover, another study also confirms the mediating effect of EE in the relationship between motivation and JS when examining the model in Indonesia’s IT industry (Riyanto et al. 2021). In the hospitality industry, one study also finds that engaged individuals tend to affect the connection between internal branding and JS positively (Lee et al. 2014). Prior research results reveal that EE also mediates the influence of organisational inducements (financial rewards and career development support) on industry loyalty (George et al. 2020). In the education sector, scholars just examine the mediating role of employee motivation in the IC-EL linkage (Nadeak and Naibaho 2020). This means that fewer studies discover the role of EE as a mediating variable in education, especially in HEIs. The present study is used to discover whether EE has any mediating effect on the IC-JS and IC-EL linkages or not, so we propose the following hypotheses, with Fig. 1 displaying the hypothesised model:

Hypothesis 5a: Job engagement mediates the relationship between internal communication and job satisfaction.

Hypothesis 5b: Organisation engagement mediates the influence of internal communication on job satisfaction.

Hypothesis 6a: Job engagement mediates the relationship between internal communication and employee loyalty.

Hypothesis 6b: Organisation engagement mediates the relationship between internal communication and employee loyalty.

## Methodology

### Data collection and sampling

Binh Duong province is located in the Southeast region of Vietnam which is also in the Key Economic Zone. With 29 industrial parks and suitable regulations, and policies from the local government, this province ranks third place in attracting foreign direct investment in Vietnam (Thai 2021). It means that the demand for the labour force is high, and education needs to be improved especially HEIs to meet the demand. Generally, there are eight universities in the province which are Binh Duong University, Thu Dau Mot University, Binh Duong Economics and Technology University, Eastern International University, Vietnamese-German University, Ho Chi Minh Open University, Thuy Loi University, and Ngo Quyen University. Four out of eight universities were selected as shown in Table 1 due to two reasons. First, these selected four universities may be considered representative of the larger population of universities in Binh Duong, covering a diverse range of institution types, sizes, and specialisations. By focusing on these four universities, the study can still yield meaningful insights applicable to the broader higher education landscape in Binh Duong province. Second, the researchers have had better access to the selected four universities due to our established relationships with these four institutions and because these universities are more open to external research collaboration.

**Table 1:** Information of universities.

	Established year	Public/Private	Source
Binh Duong University	1997	Private	(Binh Duong University 2018)
Thu Dau Mot University	2009	Public	(Thu Dau Mot University 2020)
Eastern International University	2010	Private	(Eastern International University 2023)
Binh Duong Economics and Technology University	2010	Private	(Binh Dương Economics and Technology University 2023)

Respondents from private universities account for 49% of the collected data (Binh Duong University, Eastern International University, and Binh Duong Economics and Technology University), and 51% of the respondents are from Thu Dau Mot University, a public university. This study used questionnaires to collect data from academic and non-academic staff. The questionnaire was translated into Vietnamese and then a pilot test was applied to get advice on the content, phrasing, and layout. Twenty educational employees and professionals were the respondents to this test.

### Measurement scale

There are six parts to the questionnaire including demographics, internal communication (IC), organisation engagement (OE), job engagement (JE), job satisfaction (JS), and employee loyalty (EL). A five-point Likert scale was used, ranging from 1 = strongly disagree, 2 = disagree, 3 = neutral, 4 = agree and 5 = strongly agree. Firstly, three items from Powell and Dent-Micallef (1997) were used to test IC, including open communication, interaction, and accept change. Secondly, EE was measured by JE and OE which was adapted from Saks (2006). With JE, eight items were used to measure it and OE was measured using six items from Chathoth et al. (2007), including salary, promotion, security, and decision-making. Finally, EL was measured using items from Zeithaml et al. (1996).

The survey was conducted online using a Google Form (Ha 2022b) from May to July 2022. After this particular collection was completed using the convenience sampling method, there were 265 respondents. However, during screening and testing usability and reliability, only 255 responses were found to be valid to be analysed in the next steps, yielding a response rate of 96.2%. This is considered a high response rate and reduces the likelihood of response bias (Malhotra 2020). In terms of sample size, the study by Hair et al. (2011, p. 144) states that “at least ten times the largest number of structural paths directed at a particular latent construct in the model” should be used to define the minimum sample size. In this study, there are four constructs which are OE, JE and JS directed at EL, and IC directed at JS which means that the minimum sample size is 30 participants and 265 respondents meet the requirement.

### Data analysis

The partial least squares structural equation modelling (PLS-SEM) method is applied to examine and test the hypotheses and data in this research (Ha 2022a; Ha et al. 2023). Moreover, SmartPLS 3.0 software is used to analyse data using the PLS-SEM approach (Ringle et al. 2015). According to (Hair et al. 2011), PLS-SEM is a useful statistical approach that is applied to analyse data in different fields, particularly business. Moreover, PLS-SEM can be seen as a tool for researchers to discover relationships between factors and results that can be applied to theoretical and practical implications. The PLS-SEM method is used to assess the model in the inner and outer ways. In the first step, the study by Chin and Marcoulides (1998) comments that the validity (convergent and discriminant) and reliability of variables are examined to confirm the outer model. With the final step, proposed relationships are proved by testing the inner model.

#### Findings

Table 2 portrays the demographic details of 255 respondents from several universities. In the sample, there are 128 (50.2%) males and 127 (49.8%) females, which is a fairly even mix of genders for the participants. Most of the employees are from 30 to 39 years old, which accounts for 62.4% of the total employees. Moreover, 18.4% of employees in this survey are from the 20 to 29 age group and the remaining 19.2% are above 40. In terms of marital status, 62.4% of the individuals are married, 29.4% of employees are single and the rest are divorced (8.2%). Regarding the educational background of the respondents, only 1 person has just graduated from secondary school and 2 participants have got a diploma degree. Furthermore, most of the respondents have a Master’s degree which accounts for 62.4%, and Bachelor’s degree as well (25.5%). As can be seen from the table, over 61% of the participants in this sample are from a faculty in different universities and 45.5% of employees have working experience of more than 10 years. Most people work in public universities (51.0%) compared to private universities (49%).

**Table 2:** Respondents’ demographic profile.

Gender		
Male	128	50.2%
Female	127	49.8%
Age		
20–29	47	18.4%
30–39	159	62.4%
40–49	36	14.1%
>50	13	5.1%
Marital status		
Single	75	29.4%
Married	159	62.4%
Divorced	21	8.2%
Education		
Secondary	1	0.4%
Diploma	2	0.8%
Bachelor	65	25.5%
Master	159	62.3%
Doctorate	28	11.0%
Academics		
Academic staff	157	61.6%
Non-academic staff	98	38.4%
Tenure		
1–4	76	29.8%
5–9	63	24.7%
≥10	116	45.5%
Type		
Private	125	49.0%
Public	130	51.0%

Source: Authors’ calculation.

Based on Hair et al. (2011) and Fornell and Larcker (1981), the PLS measurement model is applied to test the structure’s reliability and validity. It includes outer loadings, composite reliability (CR), average variance extracted (AVE), and the Heterotrait-Monotrait (HTMT) criterion.

### Outer model evaluation

Table 3 demonstrates relevant indicators of outer loadings, CR, Alpha’s value and AVE. It indicates that the outer loading figures are higher than 0.7 and CR ranges from 0.903 to 0.951. All of them meet the requirements by exceeding 0.7. Moreover, the AVE indicator ranges between 0.698 and 0.813 which satisfies the requirement (more than 0.5). Based on the results, the reliability and convergent validity are established.

**Table 3:** The results from the measurement model estimation.

Constructs	Code	Items	Outer loading	CR	*α*	AVE
Internal communication (IC)	IC1	Written and oral communications are very open in our company.	0.911	0.909	0.850	0.770
	IC2	Our employees communicate widely, rather than just with their own departments and functions.	0.883			
	IC3	In general, our employees accept change readily.	0.836			
Job engagement (JE)	JE1	Sometimes, I am so into my job that I lose track of time.	0.754	0.951	0.940	0.709
	JE2	I really “throw” myself into my job.	0.869			
	JE3	My mind never wanders and I do not think of other things when I’m doing my job.	0.743			
	JE4	I am highly engaged in this job.	0.923			
	JE5	The job I have makes me enthusiastic.	0.850			
	JE6	I view my job as being meaningful.	0.891			
	JE7	I am enthusiastic about the job I do.	0.906			
	JE8	When I wake up in the morning, I really want to go to work.	0.780			
Organisation engagement (OE)	OE1	Being a member of this organisation is very captivating.	0.889	0.951	0.938	0.765
	OE2	I am really into the “goings-on” in this organisation.	0.890			
	OE3	Being a member of this organisation makes me come “alive”.	0.869			
	OE4	Being a member of this organisation is exhilarating for me.	0.896			
	OE5	I am highly engaged in this organisation.	0.878			
	OE6	I am committed to this organisation.	0.823			
Job satisfaction (JS)	JS1	I am happy to work for this organisation.	0.857	0.932	0.913	0.698
	JS2	I feel that I am fulfilling an important role in the organisation.	0.850			
	JS3	I have been recognised by my peers in my efforts to contribute to the organisation’s success.	0.859			
	JS4	I am confident of my future success within this organisation.	0.881			
	JS5	I feel that this organisation pays for performance.	0.720			
	JS6	I am satisfied with the supervision level in this organisation.	0.835			
Employee loyalty (EL)	EL1	I will be happy to spend the rest of my career in this organisation.	0.886	0.929	0.885	0.813
	EL2	I say positive things about this organisation to other people.	0.909			
	EL3	I recommend this organisation to someone who seeks my advice.	0.909			

Source: Authors’ calculation.

Additionally, to evaluate discriminant validity, values of heterotrait-monotrait (HTMT) ratio were used against a threshold of 1.0 (Garson 2016). Although the highest value of HTMT is 0.946 (IC-EL), it still falls below the cut-off of 1.0 (Table 4). Moreover, none of the HTMT ratio of correlations statistics is 95 percent bias-corrected, and accelerated confidence intervals (CIs) contains the value of 1.0, supporting the discriminant validity (Franke and Sarstedt 2019) (Table 4).

**Table 4:** Discriminant validity results using HTMT.

	EL	IC	JE	JS	OE
EL					
IC	0.946				
JE	0.897	0.871			
JS	0.938	0.934	0.842		
OE	0.978	0.893	0.904	0.909	

Source: Authors’ calculation.

### Inner model evaluation

**Fig. 2:**
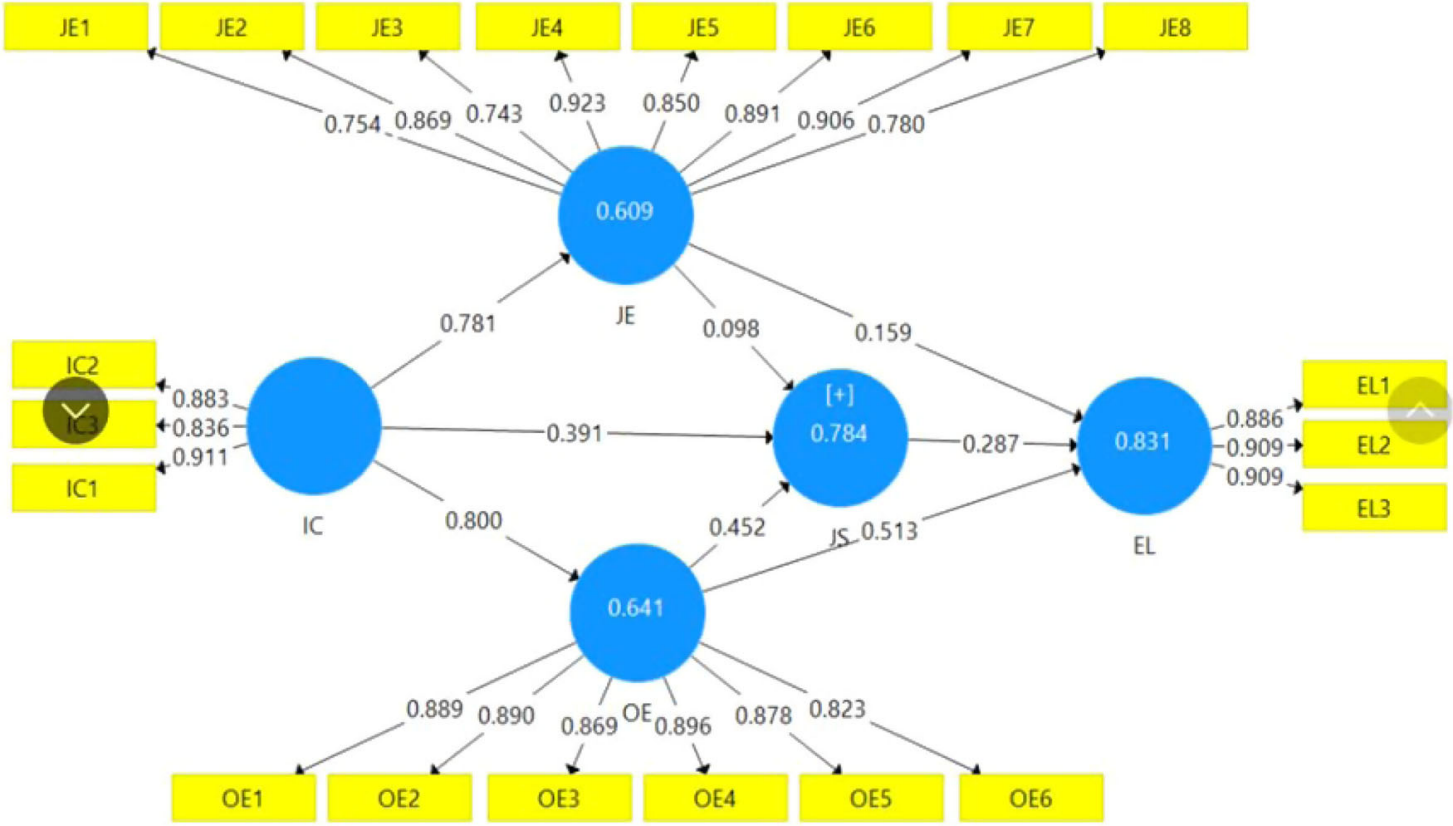
Empirical results using SmartPLS.

The study by Chin and Marcoulides (1998) demonstrates how the R-squared model depicts the variance of variables and forecasts the inner model’s accuracy. Based on Hair et al. (2010), these R-squared values have to be higher than 0.10 and significant at the 0.01 level. Moreover, these indicators can explain the level of the variables. When the R-squared is 0.67, 0.33 and 0.19, the value is substantial, moderate or weak (Chin and Marcoulides 1998). Based on the diagram, the R-square is 0.784 (substantial level), indicating that IC and EE explain the 78.4% variance of JS in the model. R-square of EL is 0.831 as a strong level so it means that the effect of independent variables like IC, EE and JS explain the 83.1% variance of EL (Fig. 2).

Cohen’s f-square is used to illustrate the effect size level of the path model, and it also highlights the changing of the R-square when a variable is removed from the model (Hair et al. 2011). Cohen (1988) says that, if its value is 0.35, 0.15 and 0.02, the effect size would be strong, moderate and weak, respectively. The table demonstrates that OE has strong effects on JS and EL, whereas JE has weak effects on both JS and EL with f-square being 0.011 and 0.038, respectively. Moreover, there is a strong effect between IC and JS (0.229). With a value of 0.129, JS and EL have a moderate effect (Table 5).

**Table 5:** Effect size analysis.

	f-square	Effect size
Effect size for job satisfaction		
IC	0.229	Strong
JE	0.011	Weak
OE	0.212	Strong
Effect size for employee loyalty		
JS	0.129	Moderate
JE	0.038	Weak
OE	0.296	Strong

Source: Authors’ calculation.

It can be seen from the Table 6 that all hypotheses are accepted except H2a. In terms of the IC-JS linkage, there is a positive impact of IC on JS with a path coefficient of 0.391 and a *p* value of 0.000. It can be concluded from these results that this relationship is significant, and H1a is supported. The standardised path coefficient value between IC-JE and IC-OE is 0.781 (*p* = 0.000) and 0.801 (*p* = 0.000), respectively, so IC has a positive effect on both JE and OE. This means that there is a significant relationship between IC and EE. Therefore, H1b and H1c are supported. JE has an insignificant effect on JS as the path coefficient and *p* value are 0.098 and 0.206, respectively. This means that H2a is unsupported. Meanwhile, there is a positive association between OE and JS (path coefficient: 0.452 and *p* value: 0.000), and it can be concluded that H3a is supported. Based on the table, there is a significant relationship between JE-EL because the path coefficients of these variables are 0.159 (*p* = 0.045). In the same fashion, the path coefficient value of OE and EL is 0.513 (*p* = 0.000), which means that OE has a positive association with EL (employee loyalty). This indicates that EE has a positive association with EL, meaning that H2b and H3b are accepted. With the standardised path coefficient value of JS and EL being 0.287 (*p* = 0.000), this proves that JS has a positive association with EL, so H4 is supported.

**Table 6:** Bootstrapping results.

Relationships	Path coefficient	Observed *T*-value	Standard deviation	Effect size (f2)	Bias	Confidence intervals (2.5%)	Confidence intervals (97.5%)	*p* value	Results
H1a: IC-JS	0.391	7.251	0.054	0.229	−0.002	0.283	0.495	0.000	Supported
H1b: IC-JE	0.781	25.507	0.031	1.559	−0.001	0.715	0.834	0.000	Supported
H1c: IC-OE	0.800	29.669	0.027	1.784	0.000	0.743	0.848	0.000	Supported
H2a: JE-JS	0.098	1.265	0.077	0.011	0.003	−0.041	0.254	0.206	Not supported
H2b: JE-EL	0.159	2.007	0.079	0.038	0.001	0.018	0.324	0.045	Supported
H3a: OE-JS	0.452	5.912	0.076	0.212	−0.001	0.298	0.597	0.000	Supported
H3b: OE-EL	0.513	5.549	0.092	0.296	0.003	0.332	0.690	0.000	Supported
H4: JS-EL	0.287	4.592	0.062	0.129	−0.005	0.157	0.403	0.000	Supported

Source: Authors’ calculation.

With indirect effects, the mediating effect’s path coefficient value of IC-OE-JS is 0.362 (*p* = 0.000), indicating that OE significantly mediates the impact of IC on JS and H5a is supported. Meanwhile, H5b is rejected, because OE insignificantly mediates the relationship between IC and JS with a path coefficient value of 0.076 (*p* = 0.213). Finally, H6a and H6B are supported with path coefficient values of 0.410 (*p* = 0.000) and 0.112 (*p* = 0.05), respectively (Table 7).

**Table 7:** Indirect effects analysis.

Relationships	Path coefficient	Observed *T*-value	Standard deviation	Bias	Confidence intervals (2.5%)	Confidence intervals (97.5%)	*p* value	Results
H5a: IC-OE-JS	0.362	5.589	0.065	−0.001	0.235	0.488	0.000	Supported
H5b: IC-JE-JS	0.076	1.246	0.061	0.003	−0.031	0.201	0.213	Not Supported
H6a: IC-OE-EL	0.410	5.134	0.080	0.003	0.257	0.569	0.000	Supported
H6b: IC-JE-EL	0.112	3.610	0.064	0.002	0.013	0.263	0.000	Supported

Source: Authors’ calculation.

## Discussion

The objectives of the present study are to identify the roles of internal communication, job engagement, organisation engagement and job satisfaction in producing employee loyalty. In addition, this study also validates the mediating role of job engagement and organisation engagement in the internal communication-job satisfaction and internal communication-employee loyalty relationships. According to the results, some hypotheses are proven significantly by the research findings, although two of them are rejected after analysing the data. First, IC has a positive effect on JS which is supported by several studies (Carrière and Bourque 2009; Ferdous and Polonsky 2014; Waiphot et al. 2018; Dhiman and Arora 2023; Pološki Vokić et al. 2023). Internal communication refers to the process of sharing information, ideas, and feedback within an organisation, fostering a transparent and collaborative working environment. Job satisfaction, on the other hand, refers to the extent to which employees are content with their jobs and work environment. An earlier study in China also refers to the positive influence between IC and JS in the context of the supply chain. Moreover, researchers assert that IC is an important factor in achieving JS and organisational success (Jacobs et al. 2016). Effective communication in the working environment positively affects the attitudes and performance of employees. Additionally, these effects result in a rise in JS within the organisation (Lee et al. 2013). By fostering a friendly and understanding workplace, effective IC can improve workers’ attitudes and performance. According to Lee et al. (2013), these favourable outcomes may boost employee job satisfaction inside the organisation. Several research in various fields and industries, including technology (Waters et al. 2013), cosmetics (Kang and Sung 2017) and cross-sectional companies (Nikolić et al. 2013), have shown this connection. The discussion highlights the fact that this study is unique in that it is the first to examine how IC has affected JS in relation to Binh Duong’s higher education institutions (HEIs). Therefore, this research addresses a critical gap in existing knowledge. The empirical results indicate that IC is essential for increasing JS among academic and non-academic staff in HEIs. The study also shows the value of IC in building relationships within the organisation, highlighting the significance of both written and oral communication. Implementing effective programmes to promote and adapt IC among employees is crucial for enhancing JS and organisational success. In short, effective IC is crucial for fostering JS and overall organisational success. By prioritising IC strategies and skill development, organisations can enhance EE, collaboration, and JS, ultimately leading to improved performance and reduced turnover rates.

Second, the empirical results that indicate a positive impact of IC on JE and OE, highlighting the significant relationship between IC and EE. EE refers to the level of commitment, involvement, and enthusiasm employees exhibit towards their work and organisation. This positive relationship between IC and EE is supported by Mishra et al. (2014) and Verčič and Vokić (2017) asserting that successful IC results in high EE, leading to an organisation’s success. This agreement between IC and EE is further corroborated by other previous studies (Welch 2011; Karanges et al. 2015; Kang and Sung 2017). Furthermore, open and honest communication within an organisation can positively promote EE and employee commitment (Riyanto et al. 2021). Highly engaged employees tend to exhibit higher productivity, retention, and JS in the workplace (Schaufeli et al. 2002). This highlights the importance of fostering a work environment that encourages open communication, feedback, and collaboration. Moreover, the discussion highlights the role of managers in improving job engagement (JE) and organisational engagement (OE). According to Mishra et al. (2014), manager involvement can significantly enhance JE and OE when they actively engage in sharing and communicating with their employees. This implies that managers should prioritise building strong relationships with their team members, fostering a culture of trust and open dialogue. In summary, the empirical results demonstrate a positive relationship between internal communication and employee engagement, with effective IC strategies leading to increased job and organisational engagement. By investing in clear communication and encouraging manager involvement, organisations can promote a highly engaged workforce, leading to improved productivity, retention, and overall success.

Third, our results indicate an interesting finding that the direct effect of JE on JS is not significant, while OE is favourably related to JS. This finding appears to contradict a previous study conducted in South Korea (Lee et al. 2014). However, it is essential to consider that differences in industries, contexts, and participants could be responsible for the varying results. Lee et al. (2014) examined the relationship between JE, OE, and JS in the hospitality context, surveying service employees working in hotels. The significant result found in their study could be attributed to the specific industry and participant group they focused on, which may differ from the context of the current study. Despite the lack of a significant direct effect of JE on JS, the empirical findings suggest that EE has a positive impact on EL. This observation is supported by previous studies conducted in various countries, such as Indonesia (Abror et al. 2020) and the United States (Book et al. 2019). EL refers to the level of commitment and allegiance employees show towards their organisation. A positive relationship between EE and EL indicates that when employees are engaged in their work and feel a strong connection to the organisation, they are more likely to exhibit loyalty, which in turn can lead to increased retention and overall organisational success. In short, the current research’s finding that the direct effect of job engagement on job satisfaction is not significant, while OE positively influences JS, highlights the complex interplay between various factors. Differences in industries, contexts, and participant groups could contribute to the contrasting results found in previous studies. The positive relationship between EE and EL emphasises the importance of fostering a work environment that promotes engagement, ultimately leading to increased loyalty, retention, and organisational success.

Fourth, the research highlights the direct impact of EE on EL, which is a crucial factor in driving an organisation’s development. As observed by Abror et al. (2020), engaged lecturers demonstrate higher levels of loyalty compared to their less engaged counterparts, ultimately contributing to the growth and success of the organisation. Moreover, this is one of the first studies to explore this relationship in Vietnam’s HEIs context which addresses a critical research gap. It also provides evidence that high EE improves the loyalty of the faculty and employees at HEIs. This study is among the first to explore this relationship within the context of Vietnam’s HEIs, addressing a critical research gap. By examining the connection between EE and EL in this unique setting, the research provides valuable insights that can inform strategies for fostering engagement and loyalty among faculty and employees in HEIs. The findings reveal that high levels of EE lead to improved loyalty among the faculty and staff, emphasising the importance of investing in initiatives that promote engagement. EE positively affects EL in the workplace, and loyal employees are more likely to remain committed to the organisation. This commitment, in turn, can lead to a reduced turnover rate, as employees are less likely to seek opportunities elsewhere (Abror et al. 2020). Lower turnover rates can result in several benefits for organisations, such as reduced recruitment and training costs, increased productivity, and greater overall stability. In short, the research underscores the direct impact of employee engagement on employee loyalty, particularly within the context of Vietnam’s HEIs. By understanding this relationship and implementing strategies to foster engagement and loyalty, organisations can benefit from reduced turnover rates, increased productivity, and overall success.

Finally, this study identifies a positive association between JS and EL, which is consistent with findings from previous studies. In Vietnam, research conducted in the healthcare and lodging sectors demonstrated that EL is an outcome of JS. Satisfied employees are more likely to continue working for and remain loyal to the organisation (Kim and Vinh 2020; Vuong et al. 2021). Similar positive links between JS and EL have been confirmed in studies conducted in Pakistan and Austria (Matzler and Renzl 2006; Hassan et al. 2013). This study goes further by exploring the mediating effect of OE in the relationship between IC and JS, which is an essential aspect of EE. The findings align with previous research in the hospitality context (Lee et al. 2014), which indicated that effective IC programs contribute to increased JS among employees and improved EE in the workplace. Moreover, the study’s findings reveal that EE plays a mediating role in the relationship between IC and EL, as both hypotheses H6a and H6b are supported. This suggests that effective IC can lead to increased EE, which in turn, influences EL positively. By understanding the mediating role of EE, organisations can better design and implement IC strategies that enhance both JS and EL. In short, this study contributes to the existing body of knowledge by examining the role of EE in the relationship between IC and EL. It reinforces the importance of fostering a positive work environment that prioritises effective IC, JS, and EE. By focusing on these factors, organisations can increase EL, which can lead to a range of benefits, such as reduced turnover, increased productivity, and overall organisational success.

### Theoretical contributions

This research provides three important theoretical contributions. The first, and possibly the most critical contribution of this study, is the combination of the development and validation of the hypothesised model based on the social exchange theory. The adequacy of applying this theory to EL studies has been carried out in the hospitality sector (Lee et al. 2014, 2016), in the enterprise sector (Leung 2008; Pace and Kisamore 2017; Yao et al. 2020), the healthcare sector (Jankelová et al. 2021) and public service organisations (Coyle-Shapiro et al. 2006). However, very few studies in EL literature apply social exchange theory in the HEI context. Our work applies social exchange theory in Vietnam’s HEI context and provides empirical evidence to add to the social exchange theory by showing different mechanisms that IC may affect EL via JE, OE and JS.

Second, unlike other research which focuses on hospitality, enterprise, healthcare and public service organisations, this work is grounded in SET to study IC which may affect EL via JE, OE and JS in the HEI context of Vietnam. Surprisingly, the relationship between JE and JS is not significant in the HEI context of Vietnam. This implies that the employees may not be well satisfied in the workplace, yet they are highly engaged with their job. This could be because the position is unique, such as a nurse working in a particular hospital as one of the emergency room staff. The work content consists of the actual tasks one is carrying out, such as a nurse’s obligation to demonstrate empathy, take blood samples and assess the general well-being of the patients. In this case, the context may not provide the nurse with a lot of job satisfaction, yet she may still be engaged in her work. This is in line with Alarcon and Lyons (2011). Our findings enrich the EL literature in the context of HEI, and sheds light on how organisations should reconsider their JE and JS practices and to implement them in the workplace.

Finally, a very rare research study examines the mediating role of EE conceptualised in both JE and OE simultaneously in the relationship between IC and EL in the HEI setting. Prior research validates EE in different sectors such as banking (Karatepe and Aga 2012), IT (Riyanto et al. 2021), or hospitality (Lee et al. 2014; George et al. 2020), enterprise sector (Leung 2008; Pace and Kisamore 2017; Yao et al. 2020), healthcare sector (Jankelová et al. 2021) and public service organisations (Coyle-Shapiro et al. 2006), except the HEI setting. The findings enrich the EL literature in the HEI context of an emerging economy like Vietnam.

### Practical contributions

Employee loyalty is a critical aspect of any organisation, including HEIs. Loyal employees are more likely to be committed, engaged, and willing to go the extra mile to support the institution’s mission and goals. To foster employee loyalty within HEIs, both individuals and university system institutions can benefit from considering the following strategies.

#### Competitive compensation

Ensuring that employees are fairly compensated for their work, in terms of salary and benefits, is crucial to fostering loyalty. HEIs should regularly review compensation packages to ensure they remain competitive within the industry (Alanezi et al. 2016; Awadallah and Elgharbawy 2021).

#### Employee recognition and appreciation

Recognising and appreciating employees for their hard work and contributions helps to create a positive work environment. This can include formal recognition programmes, regular feedback, and celebrating individual and team successes (Sghari 2016; Yang and Jiang 2023).

#### Professional development

Providing opportunities for employees to grow and develop within their roles is important for employee satisfaction and loyalty. HEIs should offer ongoing professional development and training opportunities, as well as resources for employees to develop new skills (Sona et al. 2022; Schwarzenthal et al. 2023).

#### Clear communication

Open, transparent communication within an organisation helps to build trust and loyalty. HEIs should strive for open lines of communication at all levels, sharing information about institutional goals, challenges, and successes (Drahota et al. 2016; Pham et al. 2023).

#### Shared values and goals

Employees who feel connected to the institution’s mission and values are more likely to remain loyal. HEIs should work to create a strong organisational culture that fosters a sense of purpose and shared goals (Mula-Falcón and Caballero 2022; Kolontari 2023).

#### Work-life balance

Supporting employees in achieving a healthy work-life balance is essential for their overall well-being and job satisfaction. HEIs should offer flexible working arrangements, such as remote work options and flexible hours, to accommodate employees’ personal needs (Crawford et al. 2023; Farber et al. 2023).

#### Opportunities for advancement

Employees are more likely to be loyal if they believe they have a future within the organisation. HEIs should provide clear paths for career progression and promote from within whenever possible (Hockett 2021; Lessky et al. 2022).

#### Supportive work environment

A supportive, inclusive work environment promotes employee loyalty. HEIs should foster a culture of collaboration and teamwork and ensure that employees feel valued, included, and respected (Naeem et al. 2019; Sarwar et al. 2021).

## Conclusions, limitations and future directions

### Conclusions

After analysing the data, the findings answer three research questions. First, EE, job engagement and organisation engagement all have a positive influence on EL, which means that engaged employees tend to gain loyalty with an organisation. Moreover, JS also has a significant relationship with EL. Second, the results also show the mediating role of job engagement with other constructs; i.e. the finding indicates the significant mediating role of job engagement in the relationship between IC and EL. However, with the IC-EL linkage, there is an insignificant mediating role between these constructs. Finally, organisation engagement mediates the influence of IC on JS and EL.

### Research limitations and future directions

The present study has only examined the model in the context of HEIs, and particularly in Binh Duong with faculty and non-academic staff, so it contains some limitations. The findings cannot be applied to other industries and sectors due to the unique culture and different settings of this study. Future research should examine the research paradigm in other sample sizes and diverse industries to see if there are different results. As there are only four main variables being used in this study (i.e. IC, EE, JS and EL), future researchers should add more constructs to expand the model and have new findings. Moreover, a different sampling method needs to be applied in future research instead of convenience sampling in order to make in-depth insights and findings. Future studies should modify the measurement scales of IC, EE, JS, or EL used in this study and establish new measurements based on prior studies to gain different results.

## Supplementary information

Dataset

## Data Availability

All data generated or analysed during this study are included in this published article and its Supplementary file.
